# Beneficial Effects of Exercise in Neuropathic Pain: An Overview of the Mechanisms Involved

**DOI:** 10.1155/prm/3432659

**Published:** 2025-02-25

**Authors:** Ali Ghanbari

**Affiliations:** ^1^Research Center of Physiology, Semnan University of Medical Sciences, Semnan, Iran; ^2^Department of Physiology, Faculty of Medicine, Semnan University of Medical Sciences, Semnan, Iran

**Keywords:** exercise, hypoalgesia, mechanisms, neuropathic pain

## Abstract

Neuropathic pain is a prevalent issue that often arises following injuries to the peripheral or central nervous system. Unfortunately, there is currently no definitive and flawless treatment available to alleviate this type of pain. However, exercise has emerged as a promising nonpharmacological and adjunctive approach, demonstrating a significant impact in reducing pain intensity. This is why physical therapy is considered a beneficial approach for diminishing pain and promoting functional recovery following nerve injuries. Regular physical activity exerts its hypoalgesic effects through a diverse array of mechanisms. These include inhibiting oxidative stress, suppressing inflammation, and modulating neurotransmitter levels, among others. It is possible that multiple activated mechanisms may coexist within an individual. However, the priming mechanism does not need to be the same across all subjects. Each person's response to physical activity and pain modulation may vary depending on their unique physiological and genetic factors. In this review, we aimed to provide a concise overview of the mechanisms underlying the beneficial effects of regular exercise on neuropathic pain. We have discussed several key mechanisms that contribute to the improvement of neuropathic pain through exercise. However, it is important to note that this is not an exhaustive analysis, and there may be other mechanisms at play. Our goal was to provide a brief yet informative exploration of the topic.

## 1. Introduction

Neuropathic pain is a chronic condition that often results from injuries or diseases affecting the nervous system [1, 2]. Traumatic nerve injuries—both central and peripheral—metabolic disorders such as diabetes, and chemotherapy treatments, as well as central and peripheral and central nervous system disorders (trigeminal neuralgia, stroke, and multiple sclerosis), and limb amputations are all examples of conditions that can contribute to varying degrees of neuropathic pain [3, 4]. Epidemiological studies suggest that neuropathic pain affects approximately 7%–10% of the general population [5]. Treating neuropathic pain poses significant challenges; despite the availability of pharmacological treatments, many patients find it difficult to achieve complete pain relief. Current medications often yield only partial pain relief and are associated with a range of adverse effects [6–9]. Further, the limited efficacy of these drugs and potential side effects including misuse, dependence, and negative consequences related to invasive methods (surgically implanted epidural motor cortex stimulation or acupuncture and percutaneous electrical nerve stimulation that require needling) have restricted the success rate of pharmacological treatments [10]. Moreover, the complexity and evolving nature of neuropathic pain characterized by diverse underlying mechanisms that change over time has imposed more challenges in the treatment of neuropathic pain so far [11]. In light of these challenges, nonpharmacological therapies may serve as valuable adjuncts in pain management and could enhance the effectiveness of pharmacological strategies.

Research on exercise-induced hypoalgesia (EIH) dates back several decades, with significant studies emerging in the early 2000s. This ongoing research highlights the complex interplay between exercise and pain modulation, suggesting that exercise can lead to significant reductions in pain perception through various physiological mechanisms. The relationship between exercise and neuropathic pain has gained significant attention in recent research, highlighting exercise as a potential therapeutic intervention. Various forms of exercise, including aerobic and resistance training, have demonstrated efficacy in alleviating neuropathic pain symptoms in both clinical and preclinical studies [12]. Exercise has been widely recognized as a nonpharmacological approach that can significantly improve the quality of life, both in healthy individuals and those dealing with various health conditions [13–16]. Numerous studies have suggested that exercise can have a hypoalgesic (pain-reducing) effect in both animals and humans [6, 17–21]. Physical activity and exercise play a crucial role in the management of neuropathic pain, especially considering the incomplete success of prescribed medications in providing complete relief [22–29]. Given the incomplete success of prescribed drugs in providing complete relief for neuropathic pain, the importance of physical activity and exercise becomes even more significant. A lot of studies in animals and humans have shown the hypoalgesic effect of aerobic exercise with different intensities and durations in animals and humans [30–35]. It has been shown that anaerobic exercise, such as aerobic exercise, has positive effects in reducing neuropathic pain. Several reports showed that strength and endurance training reduce oxidative stress, prevent proinflammatory cytokine production, increase deep dorsal horn neuronal plasticity, and decrease chronic neuropathic pain in autoimmune encephalomyelitis mice [36–38]. On the other hand, there are conflicting reports about resistance or isometric EIH which is important clinically [35, 39–41]. Resistance training, when compared to aerobic exercise, hinders functional recovery and does not affect the regeneration of sciatic nerve fibers [42]. It has been demonstrated that isometric exercise lowers the pain rating in humans with a more significant reduction in heat pain observed in women compared to men [40]. There is a limited amount of research available on isometric exercise in animals, which may be attributed to technical problems in conducting animal studies on isometric exercise. However, the studies reveal that almost most researchers believe that anaerobic exercise, like aerobic exercise, effectively reduces neuropathic pain through somewhat similar mechanisms.

Although several exercise techniques have been shown to alleviate neuropathic pain, there is not a single mechanism of action. Various mechanisms are involved in EIH, and the present review attempts to address the most common ones. Most of the information on neuropathic pain has been obtained from different animal models developed to simulate human neuropathic pain.

### 1.1. Search Strategy

Materials were searched from PubMed and Web of Science reference databases. Key search terms included, neuropathic pain, sciatic nerve injury, peripheral neuropathy, thermal hyperalgesia, allodynia, physical activity, treadmill exercise, and swimming exercise. The searched studies were reviewed manually for other potentially relevant citations. English language papers were used.

Original studies (human and animal) particularly were used. The evaluated papers investigated the effects of exercise on neuropathic pain in rodent models induced by nerve injury.

Included matters were neuropathic pain in rat models induced by peripheral nerve injury (induced by ligation, constriction, and diabetic neuropathy) and central nervous injury (spinal cord injury).

### 1.2. Oxidative Stress

Oxidative stress is recognized as a mechanism in the development of neuropathic pain [43, 44]. The nervous system contains a substantial amount of phospholipids, making it prone to oxidative stress [45]. Exercise has been shown to have positive effects on tissues by enhancing antioxidant enzyme activity, reducing oxidative damage, and improving tissue resilience against free radical attacks in male rats [46]. Studies on human and rats have also demonstrated the ability of aerobic exercise to suppress lipid peroxidation processes [47–49]. There have been studies indicating that aerobic exercise can increase the activity of antioxidant enzymes such as superoxide dismutase (SOD), glutathione peroxidase (GPx), and catalase (CAT) in rats and mice [50, 51]. It has been observed that reactive oxygen species (ROS) in people's muscles can modulate the activities of antioxidant enzymes by activating specific signaling pathways [52].

Studies on human and animal subjects have shown that a decrease in glutathione levels can be attributed to oxidative stress, while the increase in glutathione induced by physical exercise is considered an indication of enhancement in the antioxidant system [53, 54]. Although the basal level of glutathione in rats did not change with moderate treadmill exercise, it has been reported that decreased glutathione levels following buthionine–sulfoximine administration could be reversed by engaging in moderate treadmill running [55]. A study by Moflehi et al. on healthy males showed that both medium-intensity exercise and high-intensity exercise lead to oxidative stress and increase malondialdehyde (MDA) levels [56], while contrary to this report, Wang and Huang reported that high-intensity exercise but not medium-intensity exercise leads to oxidative stress (reduces GSH level) in sedentary men [57]. The conflicting results of these studies could be ascribed to different markers assessed for oxidative stress status which may respond differentially to exercise intensities. In our previous studies, we demonstrated that the total antioxidant capacity of plasma, which was reduced in rats with neuropathic pain, significantly improved following a 3-week treadmill exercise regimen [30]. Furthermore, we found that trigeminal neuropathy led to a decrease in the antioxidant enzyme GPx, and this decrease was reversed toward the control level after 2 weeks of swimming exercise [31]. In addition, it has been reported that exercised rats exhibited a significant increase in hepatic GSH levels and GPx activity [58].

Preclinical and clinical studies have revealed that while acute exercise can temporarily increase ROS production, regular exercise has been shown to reduce the occurrence of oxidative stress-related disorders. Acute exercise increases muscle nociceptor sensitization through activation of ROS production and promotion of the redox state [59]. On the other hand, regular exercise activates M2 macrophages to secret anti-inflammatory cytokine interleukin-10 (IL-10) and also through upregulation of antioxidants (PGC 1a, sirtuin-3 [SIRT3], SOD, CAT, GPx, and GSH) in the myocytes which reduces ROS production and leads to pain suppression [59]. There are conflicting findings concerning the impact of short-term exercise on pain. Some clinical research indicates that such exercise can either be painful [60, 61] or produce a temporary reduction in pain sensitivity [62, 63]. This discrepancy may arise from the various chronic pain conditions that engage different physiological mechanisms.

**Figure 1:**
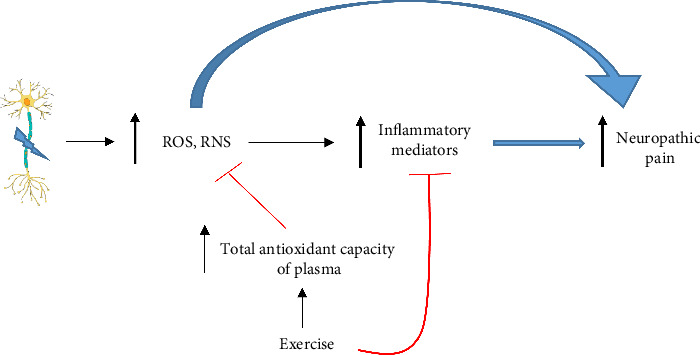
Effect of exercise on neuropathic pain induced by nerve injury. Increased ROS production following nerve injury directly and indirectly (through triggering inflammatory mediators) leads to neuropathic pain, and exercise through inhibition of ROS and inflammatory mediators prevents neuropathic pain.

Several animal studies have demonstrated that regular exercise can ameliorate oxidative stress damage [64–66]. Although exercise has many beneficial effects, it has been reported that, 3 months of high-intensity exercise, unlike moderate intensity, significantly increased serum MDA concentration in young men [67]. Furthermore, reports indicate that severe exercise can lead to an increase in ROS production beyond the capacity of the body's antioxidant system to defend against them [68–70]. Oxidative stress is considered as an inflammatory stimulus. Oxidative stress through cell death can trigger inflammatory mediator activation [71]. It should be noted that in addition to a reduction of oxidative stress, exercise suppresses the inflammatory process stimulated by oxidative stress (Figure 1).

In summary, oxidative stress plays a significant role in the development of neuropathic pain, and exercise can modulate this process. While acute exercise may temporarily increase ROS and exacerbate pain sensitivity, regular physical activity enhances antioxidant enzyme activity and reduces oxidative damage, ultimately contributing to pain relief. The effects of exercise on oxidative stress and pain perception vary depending on intensity, frequency, and duration, highlighting the complexity of these interactions in both clinical and preclinical settings. However, it is important to remember that personal characteristics, including individual differences, history, and gender, should not be neglected when considering the impact of exercise on oxidative stress.

### 1.3. Neurotransmitter Release

The role of the opioid system in reducing neuropathic pain has long been suggested as a mechanism of exercise hypoalgesia in patients [72–74]. Postexercise activation of the opioidergic system in both male and female individuals appears to be acute and is intensity-dependent [20, 75]. An important point is that in inflammatory state as neuropathic pain, exercise (in rats) not only acutely activates endogenous opioid release but also maintains the system's active for longer periods [62]. Therefore, regular exercise may lead to long-term changes in endogenous opioid release. On the other hand, it has been shown that resistance training exercise in cancer neuropathic pain patients results in immediate EIH and is mediated by the release of beta-endorphins which act as natural pain relievers by binding to opioid receptors in the brain and spinal cord, part of the descending pain pathway [76].

**Figure 2:**
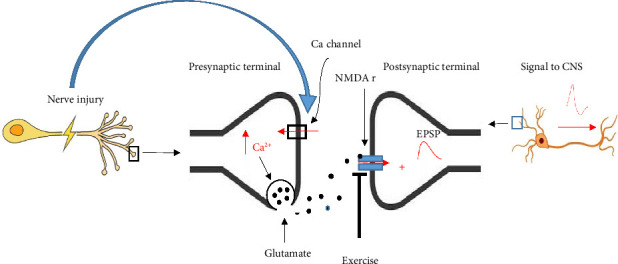
Effect of exercise on pain signals following nerve injury-induced neurotransmitter release. Nerve injury increases Ca ^2+^ concentration in presynaptic terminals by increasing Ca ^2+^ channel activity on presynaptic buttons which in turn increases glutamate release. Glutamate affects NMDA receptors on postsynaptic neurons and in turn increases nerve signals to the spinal cord and brain. Exercise through decreasing phosphorylation of the NR1 subunit of NMDA receptors prevents stimulation of postsynaptic neurons and then decreases pain signals.

Several reports indicate that neurotransmitters are involved in neuropathic pain rats [77–79]. While changes in neurotransmitter release are not the main mechanism of neuropathic pain, however, they modulate the pain to some extent. Neuronal injury through upregulation of *α*2*δ* subunit of calcium channels on the central terminal of primary neurons leads to increased Ca^2+^ entry and glutamate release ultimately resulting in neuropathic pain signals in neuropathic pain rats [80]. In clinical studies, pregabalin, through inhibition of *α*2*δ* subunit of presynaptic voltage-dependent calcium channels in the brain and spinal cord, decreases the release of excitatory neurotransmitters [81, 82] and exerting a GABA-like effect, thereby having a hypoalgesic effect on neuropathic pain [83, 84]. Previously, we demonstrated that glutamate release significantly increased following spinal cord injury in neuropathic male rats [77]. Further, we recently showed that swimming exercise significantly decreased CSF glutamate levels in sciatic-injured neuropathic pain rats [32]. Several studies have indicated that exercise modulates glutamatergic function. It has been reported (studies on rats and mice) that forced running exercise suppresses phosphorylation of the NR1 subunit of central NMDA receptors and consequently decreases pain signal transmission [85, 86]. In addition, swimming exercise has been found to reduce glutamate-induced pain in mice [87]. Briefly, it appears that nerve injury through presynaptic mechanisms increases nerve signals to the spinal cord and brain, while exercise postsynaptically modulates these signals (Figure 2).

Catecholamine (noradrenaline) plays a role in reducing pain through activation of presynaptic and postsynaptic *α*_2_-adrenergic receptors. Increased activation of the autonomic nervous system in exercised mice leads to noradrenaline release, which exerts descending antinociception [88].

Regarding preclinical studies, it has been proposed that neuromodulators such as cannabinoids play a role in nociception [89, 90]. Daniel Martins and his colleagues reported that administration of AM 281, CB1 receptor antagonist, before exercise inhibited EIH in male mice [87].

Some studies (clinical and preclinical) suggest that neurotransmitters such as serotonin and norepinephrine play a significant role in the modulation of neuropathic pain [91–93]. Bobinski and his colleagues demonstrated that decreased serotonin levels and increased serotonin transporter expression in the brain stem of neuropathic pain mice were reversed following 14 days of treadmill exercise [29]. They also showed that blocking serotonin synthesis using *para*-chlorophenylalanine methyl ester (PCPA) reduced exercise-induced analgesia in neuropathic pain mice.

Some reports indicate that physical activity prevents the increased expression of the serotonin transporter in the rostral ventromedial medulla of nerve-injured animals [94] leading to increased serotonin release and activity. In general, both aerobic exercise and resistance exercise enhance the activity of catecholamine, opioid, and cannabinoid systems, which raises the pain threshold.

### 1.4. Neurotrophic Factors

In vivo (animal models) and in vitro studies have shown that neurotrophins (NTs) (neurotrophic factors) regulate the growth, maintenance, and apoptosis of both intact and injured neurons [95–97]. NTs including nerve growth factor (NGF), brain-derived neurotrophic factor (BDNF), NT-3, and NT-4 have similar structures and functions [95, 96].

Studies on rats and mice showed that NGF, the first member of the neurotrophin family, plays a neuroprotective role in neuronal development, function, and the repair of damaged peripheral nerves [98, 99]. Further, administration of NGF in patients with acute ICH (intracerebral hemorrhage) improved neurological functions and reduced disability [100].

It has been reported that exogenous NGF promotes the removal of myelin debris by Schwann cells in sciatic nerve-injured rats significantly increasing the speed of remyelination of damaged nerves [101].

Animal studies on the beneficial effects of exercise on neurotrophic factors show that exercise increased NT synthesis [102] and improved signaling of neurotrophic receptors in both the central and peripheral nervous systems [28, 103–107]. Physical activity positively affects injured peripheral neurons and functional recovery through alterations in NT expression [108].

Moreover, in humans and rodents, physical activity induces BDNF, a NT, associated with neuronal plasticity [109, 110]. Research on humans has shown that the concentrations of NTs, especially BDNF, rise significantly as a result of exercise [111] and this increase is influenced by exercise intensity [112]. Almeida and colleagues reported that aerobic exercise increases BDNF levels while decreasing mechanical sensitivity in a neuropathic pain mice model [26].

Contrary to reports regarding the antinociceptive role of NTs, the study by Lopez–Alvarez and colleagues has shown that NGF and BDNF have a pronociceptive role; thus, exercise suppresses pain in neuropathic pain rats by decreasing these neurotrophins in sensory neurons, [113]. However, it seems that factors such as the type, duration, and intensity of exercise contribute to these controversies and these should be taken into consideration when interpreting research results.

In summary, NTs, such as NGF and BDNF, regulate neuronal growth, maintenance, and repair. While NGF has neuroprotective effects in peripheral nerve injury and exercise increases NT synthesis, studies show conflicting roles of NTs in pain modulation. Specifically, NGF and BDNF may promote pain under certain conditions, suggesting that exercise can suppress pain by reducing these NTs in sensory neurons. Factors such as exercise type, duration, and intensity significantly influence these outcomes.

### 1.5. Inflammatory Process

Nerve injuries promote the expression and production of inflammatory mediators, including cytokines such as tumor necrosis factor (TNF)-*α* and IL-1β in rodents [30, 114, 115].

Several studies suggest that exercise modulates the inflammatory and immune response in rodents, to restore cytokines and neuroimmune mediators in the central and peripheral nervous system to preinjury levels [27, 77, 103, 107, 116–119]. Moreover, it has been shown that proinflammatory cytokines decrease while anti-inflammatory cytokines increase in exercised human subjects [120–122]. Exercise, especially aerobic, is capable of reducing the effects of inflammatory reactions in chronic diseases such as diabetes [123].

It has been reported that exercise reduces microglial cell activation and cytokine release through sympathetic system activation, which in turn alleviates neuropathic pain [124].

Previously, we showed that increased levels of cerebrospinal fluid TNF-*α* in neuropathic male rats significantly decreased following 3 weeks of treadmill exercise [30].

Accordingly, Yu-Wen Chen and his colleagues reported that 21 days of swimming or treadmill exercise significantly reduced TNF-*α* and IL-1β cytokines in the sciatic nerve of neuropathic pain rats [125]. It is important to mention that increased cytokine levels can induce microglial cell activation to release more cytokines and excitatory amino acids, leading to a vicious circle that promote persistent neuropathic pain. Therefore, exercise as an anti-inflammatory approach plays a prominent role in suppressing neuropathic pain.

### 1.6. Gene Expression

Various studies have shown that regular physical activity changes gene expression [126]. Exercise after nerve injuries leads to the expression of genes involved in axonal outgrowth. Nahid and her colleagues reported that fibronectin gene expression decreased in the sciatic nerve of rats with diabetic neuropathy and aerobic exercise increased it to the control level [127]. In parallel, fibronectin protein changed in line with gene expression changes. Ring finger protein 34 (RNF34) gene increases in dorsal horn neurons of neuropathic pain rats and is suppressed following exercise [128]. In vivo and in vitro studies have revealed that RNF34 decreases the density of dorsal horn GABA_A_ receptors, while exercise increases GABA_A_ receptors through inhibition of *RNF34* gene expression, leading to pain relief [129].

Pituitary adenylate cyclase-activating polypeptide (PACAP) is a pain-inducing polypeptide. Intrathecal administration of PACAP38 in mice leads to pain-like behavior [130]. It has been shown that 3 weeks of aerobic exercise decreased spinal cord PACAP mRNA levels and, in parallel, decreased neuropathic pain in spinal nerve-ligated rats [128]. Mabuchi et al. reported that, unlike wild-type mice, the application of NMDA in mice lacking the Pacap gene (Pacap ^_^/^_^) does not result in mechanical allodynia [131]. This suggests a relationship between NMDA and PACAP gene expression. It should be mentioned that NMDA receptor activation decreases following exercise [85, 86]. Fuccio et al. reported a correlation between allodynia and hyperalgesia and overexpression of IL-1β and caspases (caspases 1, 8, and 12) genes in the spared nerve injury (SNI) mice brain [132]. On the other hand, there are reports about the decreasing effects of exercise on proinflammatory and apoptotic gene expressions in rodents [133, 134]. Exercise increases BDNF and NT3 mRNAs and promotes nerve regeneration in peripherally injured nerve rats [135, 136]. This confirms the possibility that neural regeneration is a process dependent on the subject's activity. Regarding these reports, it seems that gene therapy can be a promising method to improve neuropathic pain conditions, as several new reports on gene therapy in animal models have shown valuable results [137–139].

Regular physical activity significantly influences gene expression related to nerve regeneration and pain modulation. Exercise enhances the expression of beneficial genes while suppressing those associated with neuropathic pain, such as RNF34 and PACAP. These findings suggest that gene therapy may be a promising approach for improving neuropathic pain conditions, supported by emerging evidence from animal models. It is important to note that considerable future research is necessary to further explore gene therapy and its potential to enhance therapeutic applications in clinical settings.

## 2. Conclusion

The beneficial effects of exercise on health and disease are a well-established topic. Generally, pain relief following acute exercise seems to occur soon but temporarily, likely through activation of opioidergic, endogenous cannabinoid, and catecholaminergic systems. However, regular exercise induces both short-term hypoalgesia and long-lasting hypoalgesia which occur through mechanisms for short-term hypoalgesia mentioned above, as well as histological changes including receptor phosphorylation and receptor upregulation in different relays of pain matrix and neuronal plasticity. Briefly, the mechanisms involved in the hypoalgesic effect of exercise in neuropathic pain are listed in Table 1.

While various mechanisms are known to contribute to EIH, it remains unclear which mechanism initiates the process, the extent of each mechanism's contribution to hypoalgesia, and which mechanism plays a key role in EIH. In addition, it is uncertain whether all mechanisms are involved in every subject or if their involvement depends on factors such as subject status, type, location, and intensity of injuries. Further, it is not clear how many days after the injury, exercising has more positive effects, and whether the timing to start exercising after the injury in examined animals is similar to that in humans or it needs to be equalized. Moreover, it is not clear whether the duration of exercise in experimental animals is sufficient for humans or whether it should be equated to life span. These questions are gaps in the available information that require further clarification and should be studied in future research.

It is conceivable that different mechanisms may interact to some degree, and these interactions may occur at different time points. According to Table 2, factors such as the type, intensity, duration, and timing of exercise following nerve injury, as well as the type of tissue, time, and method of experimental assay, are crucial parameters that must be considered when interpreting data. Furthermore, the characteristics of the subjects under study, particularly their sex, psychological state, gender, and lifestyle history, can significantly impact the results. Nonetheless, it is clear that exercise at submaximal intensities has a hypoalgesic effect, making it a viable adjunctive treatment option for patients experiencing chronic pain, particularly neuropathic pain.

A review of the literature indicates that many animal studies fail to adequately account for the psychological state of the subjects, complicating the translation of findings to humans. On the other hand, despite the differences between sexes in various factors such as hormones and task abilities, most animal studies have been conducted on male subjects, whose findings are often applied to both human sexes. To draw more reliable conclusions, comparisons of results should be based on similar experimental conditions and sample populations. This approach will help ensure the validity and generalizability of the findings and develop an applicable formulation of exercise for neuropathic pain.

Finally, most of the available information comes from animal studies that cannot be exactly extrapolated to humans. Given reports regarding the potentially stressful nature of intense exercise and the limited effectiveness of low-intensity exercise, medium-intensity exercise—whether aerobic, isometric, or resistance training—has proven valuable effects in reducing pain sensitivity in conditions characterized by neuropathic pain.

### 2.1. Future Research

Some important questions should be answered in future studies including whether an exercise is suitable for both males and females; what the most suitable type of exercise is for neuropathic pain with different etiology, and which is more hypoalgesic, aerobic or strengthening exercise, at the same intensity for the same duration in treating the same neuropathic pain conditions.

## Figures and Tables

**Table 1:** Mechanisms of exercise-induced hypoalgesia in different types of exercise.

Type of exercise	Pain threshold	Mechanisms	References
Isometric	Decrease	Endocannabinoid	Koltyn et al. [41]
Acute short-time	Decrease	Opioid + nonopioid	Stagg et al. [62]
Running wheel	Decrease	Opioid + inflammatory mediators + neurotransmitter system (catecholaminergic)	Dunn et al. [92]; Grace et al. [103]
Aerobic exercise	Decrease	Opioid system + oxidative stress + neurotransmitter system (catecholaminergic) + inflammatory mediators + neurotrophic factors + gene expression	Stagg et al. [62]; Almeida et al. [26]; Safakhah et al. [30]; Rostami et al. [31]; Azarbayjani and farzanegi [127]

**Table 2:** Factors that affect the results of the exercise.

Exercise	Aerobic, anaerobic
Duration	Acute, subchronic, chronic (number of weeks)
Intensity	Mild, medium, high
Timing	Daily, weekly (time per day or week)
Tested samples	Blood, CSF, tissue
Sex	Male, female

*Note:* CSF, cerebrospinal fluid.

## Data Availability

Data sharing is not applicable to this article as no datasets were generated or analyzed during the current study.
